# Oral anticoagulant and antiplatelet drugs used in prevention of cardiovascular events in elderly people in Poland

**DOI:** 10.1186/1471-2261-12-98

**Published:** 2012-10-31

**Authors:** Beata Labuz-Roszak, Krystyna Pierzchala, Michal Skrzypek, Marta Swiech, Agnieszka Machowska-Majchrzak

**Affiliations:** 1 Department of Neurology, Medical University of Silesia, 3-go Maja 13/15, Zabrze, 41-800, Poland; 2 Department of Biostatistics, Faculty of Public Health, Medical University of Silesia, Katowice, Poland; 3 International Institute of Molecular and Cell Biology, Warsaw, Poland; 4 Laboratory of Genomic Medicine, Department of General, Transplant and Liver Surgery, Medical University of Warsaw, Warsaw, Poland

**Keywords:** Epidemiology, Elderly, Public health, Preventive medicine, Oral anticoagulant drugs, Antiplatelet drugs, Acetylsalicylic acid

## Abstract

**Background:**

In Poland, the prevalence of cardiovascular diseases is increasing. This might be associated with the constantly growing proportion of elderly people and inappropriate cardiovascular prevention. This study aimed to evaluate the frequency of use of oral antiplatelet (OAP) and oral anticoagulant (OAC) drugs among older people in Poland and to assess their association with cardiovascular risk factors.

**Methods:**

The study was based on data collected during the implementation of a multicentre, publicly funded research project called PolSenior.

**Results:**

The study group consisted of 4,979 people with the average age of 79.35 ± 8.69 years. Among them, 1,787 people (35.9%) used at least one drug in the prevention of cardiovascular diseases. OAPs were used regularly by 1,648 (33.1%) elderly people and OACs were used by 165 elderly people (3.3%). Acetylsalicylic acid was used by 32.2% of elderly people. Use of drugs significantly depended on age (p < 0.01), sex (p < 0.01), place of residence (p < 0.001), level of education (p < 0.0001) and personal income (p < 0.0001). Among all the respondents treated with OAPs, therapy was applied as secondary cardiovascular prevention in 717 respondents (43.5%), and as primary prevention in 705 respondents (42.8%). Among the respondents treated with OACs, 117 (71%) elderly people had a history of atrial fibrillation. Secondary cardiovascular prevention should be considered in a further 482 respondents (15.1% of untreated elderly people), and primary cardiovascular prevention in 1,447 respondents (45.3%).

**Conclusions:**

Our study is the first to determine the frequency of use of OAP and OAC drugs among elderly people in Poland in relation to cardiovascular risk factors. The most commonly used drug for cardiovascular prevention is acetylsalicylic acid, but it appears that it is used too rarely in high-risk patients. Educational programs should be developed among general practitioners concerning current recommendations for pharmacological cardiovascular prevention.

## Background

Cardiovascular disease is the most common cause of mortality in elderly people worldwide, reaching 17 million annually [1]. The incidence of stroke, as well as myocardial infarction, increases dramatically with age, and age is the strongest risk factor for cardiovascular diseases. Although in Western European countries the incidence of coronary heart disease and stroke has been decreasing since the 1970s, in Poland and other Eastern European countries the prevalence of cardiovascular disease is still increasing [2,3]. This might be associated with the constantly growing proportion of elderly people and inappropriate cardiovascular prevention.

The most popular drugs used worldwide in prevention of cardiovascular incidents are antiplatelet agents. One of these agents is acetylsalicylic acid (ASA), registered in 1897 by Felix Hoffman as aspirin [4]. Initially, ASA was used as an anti-inflammatory, antipyretic and analgesic drug [5]. The antithrombotic effect of ASA was discovered in the 1960s, and since then, the drug has been widely used in primary or secondary prevention of cardiovascular diseases [6,7]. The occurrence of myocardial infarction and stroke has been reduced by 25% in patients regularly taking ASA [8]. Other oral antiplatelet (OAP) drugs are recommended to people with contraindications to ASA (e.g., ticlopidine, clopidogrel, and dipyridamole) [9].

Another group of medications used in prevention of cardiovascular incidents are oral anticoagulant (OAC) drugs, such as acenocoumarol and warfarin. These agents block the receptors responsible for the binding of vitamin K necessary for the normal blood clotting process [10]. These drugs are especially recommended for stroke prevention in people with atrial fibrillation, with the presence of thrombus in the left ventricle, after anterior myocardial infarction, and in some other specific clinical situations [10].

No studies have assessed the frequency of use of cardiovascular preventive therapy in elderly people in Poland or in other Eastern European countries. Therefore, this study aimed to evaluate the frequency of use of OAP and OAC drugs among older people in Poland and to assess its association with cardiovascular risk factors.

## Methods

### Description of the project

This study was based on data collected during the implementation of a multicentre, publicly funded research project commissioned by the Polish Ministry of Science and Higher Education called “Medical, psychological, sociological and economic aspects of aging of people in Poland (PolSenior)” (PBZ-MEiN-9/2/2006). The project was carried out during 3 years (from October 2008 to October 2010). The total number of participants was 5,695 (2,899 males and 2,796 females), including 4,979 people aged 65 and older, and 716 people at the threshold of old age (55-59 years). Research participants were randomly recruited in bundles, in a stratified, proportional draw performed in three stages, as described previously [11]. Each participant was asked to complete a questionnaire consisting of two parts: medical and socioeconomic. The medical part of the questionnaire included detailed questions about their present health status, as well as history of diseases, hospitalisations, and current medications. The socioeconomic part of the questionnaire included questions concerning the personal and family situation, economic status, household structure, leisure activities, hobbies, and social life, among others. English versions of the medical and socioeconomic questionnaires are available on-line (http://polsenior.iimcb.gov.pl/en/questionnaire). The medical part of the questionnaire was completed during the first visit, while the socioeconomic part was completed during the second visit. When the participant was unable to answer the questions, a caregiver was asked. During the third visit, blood and urine samples were taken and delivered within 2 h to local laboratories. In all the participants, body weight and height were also measured, and the body mass index (BMI) was calculated. Arterial blood pressure and pulse were measured three times (during each visit) by automatic blood pressure monitors (A&D UA-787 Plus, validated by the British Hypertension Society). Each measurement was performed with the participant in a seated position, on the right upper arm, after at least 5 min of rest and at 2-min intervals.

The PolSenior project was approved by the Bioethics Commission of the Medical University of Silesia in Katowice. Before enrolment in the study, each respondent or their caregiver signed an informed consent form. A detailed description of the project has been previously described [11].

### Study group

The study group included respondents from the PolSenior project aged 65 years and older.

### Data analysis

The frequency of use of drugs was based on the data collected in the medical part of the questionnaire, including the international and trade names of drugs taken by participants.

The following data from the general PolSenior database were also used and analysed: age, sex, place of residence (village or city, or staying at a nursing home), education, professional status, personal income, and a history of cardiovascular risk factors (coronary heart disease, previous myocardial infarction, congestive heart failure, pharmacologically treated hypertension, a history of atrial fibrillation, previous strokes, pharmacologically treated diabetes, pharmacologically treated dyslipidemia, and active smoking). Measurements of BMI, mean systolic blood pressure, and blood tests (total cholesterol) were also analysed.

Statistical data analysis was performed using SAS version 9.2 (SAS Institute Inc., Gary, NC). The level of statistical significance was p ≤ 0.05. Diversity variables were assessed based on the chi-square test or Fischer’s exact test. We used the Kolmogorov-Smirnov test (N > 2000) or Shapiro-Wilk test to check the compatibility of the distribution of quantitative variables with normal distribution. In the case of normal distribution, statistical significance of differences between quantitative variables was analysed using the Student’s *t*-test. When the distribution diverged from normal, the nonparametric Wilcoxon rank sum test was used. A model of logistic regression was used to analyse the association between cardiovascular risk factors and treatment with OAP and/or OAC drugs.

The 10-year risk of fatal cardiovascular disease (Systematic Coronary Risk Evaluation [SCORE]) was calculated by using the following data: sex (male/female), age (years), systolic blood pressure (mmHg), total cholesterol (mmol/l) and history of current smoking [12].

The CHADS_2_ score for estimating the risk of stroke in patients with atrial fibrillation was calculated by adding together points for congestive heart failure (1 point), hypertension (1 point), age ≥75 years (1 point), diabetes mellitus (1 point), and previous stroke (2 points) [13].

## Results

The study group consisted of 4,979 people aged from 65 to 104 years, including 2,567 men (51.56%) aged from 65 to 104 years and 2,412 women (48.44%) aged from 65 to 104 years. The mean age of the study group was 79.35 ± 8.69 years, the mean age of men was 79.48 ± 8.54 years, and the mean age of women was 79.22 ± 8.86 years. The age difference between men and women was not statistically significant (p = 0.21).

Among all the respondents, 1,787 people (35.9%) used at least one drug in the prevention of cardiovascular diseases. OAPs were regularly used by 1,648 respondents (33.1%); 1,556 of them (31.3%) were taking one drug, 90 subjects (1.8%) were taking two drugs, and two of them (0.04%) were taking three drugs. OACs were regularly used by 165 people (3.3%). Antiplatelet and anticoagulant therapy was simultaneously applied by 26 respondents (0.5%) (Table 1).

**Table 1:** Frequency of use of particular medications in the study group

	**International drug name**	**Number of respondents using the drug n (%)**
OAP	acetylsalicylic acid	1,604 (32.2%)
	clopidogrel	57 (1.14%)
	ticlopidine	81 (1.6%)
OAC	acenocoumarol	158 (3.2%)
	warfarin	18 (0.4%)

*OAP* - Oral antiplatelet drugs, *OAC* - Oral anticoagulant drugs.

With regard to other drugs applied in the treatment of cardiovascular diseases, beta-blockers were used by 1,386 (27.8%) respondents, ACE inhibitors were used by 1,973 (39.6%) respondents, angiotensin II receptor blockers were used by 343 (6.9%) respondents, calcium channel blockers were used by 141 (2.8%) respondents, and statins were used by 1,158 (23.3%) respondents.

The percentage of women using OAP and/or OAC drugs (34.3%, n = 828) was significantly lower than the corresponding percentage in men (37.4%, n = 959) (p < 0.01). The frequency of use of OAP and/or OAC drugs was statistically significantly dependent on age in women (p < 0.01), men (p < 0.01), and all subjects (p < 0.01). Drugs were most frequently taken in the age group of 80-84 years, and were most rarely taken in the youngest age group (65-69 years).

Inhabitants of cities used OAP and/or OAC drugs significantly more frequently (37.8%, n = 1130) than rural residents (33.1%, n = 656) (p < 0.001).

We observed significant differences in the frequency of OAPs and/or OACs use, depending on the province where the respondents lived (p ≤ 0.05). Preventive cardiovascular therapy was mostly applied by people from Swietokrzyskie (46.6% of respondents living in this region), Kujawsko-Pomorskie (40.7%), and Wielkopolskie (40.1%), while the lowest amount was applied by people living in Zachodniopomorskie (30.1%), Podlaskie (30.2%), Podkarpackie (31.97%), and Lubuskie (31.97%).

The frequency of use of OAP and/or OAC drugs was associated with the level of education (p < 0.0001). People who were better educated used these drugs more often. A total of 39.4% of respondents who had graduated from university applied the examined drugs, while only 20.2% of people who declared a lack of education.

Personal income had a significant effect on the frequency of use of OAP and/or OAC drugs (p < 0.0001). Only 22.4% of people with the lowest income (up to 500 Polish zloty per month) used these drugs compared with 44.7% of those with the highest incomes (2501 or more Polish zloty per month).

Professional activity of the study group did not affect the frequency of prevention of cardiovascular disease. The vast majority of responders were pensioners (87.9%, n = 4222), and among them, 36.5% had used OAP and/or OAC drugs. Eleven individuals declared unemployment (0.2%), and only one of them (9.1%) applied pharmacological prevention of cardiovascular diseases. A total of 108 respondents (2.3%) were housewives and 28.7% of them used OAP and/or OAC drugs. Active professional people constituted less than 1% of all respondents (0.9%, n = 44) and 38.6% of them used one of the examined drugs.

There were 50 residents of nursing houses (1.1%) and 21 of them (42%) had used OAP and/or OAC drugs.

The frequency of cardiovascular risk factors in the whole study group is shown in Table 2. The association between use of OAP and/or OAC drugs and cardiovascular risk factors is shown in Table 3.

**Table 2:** Frequency of cardiovascular risk factors in the whole study group (N = 4979)

**Risk factor**	**n (%)**	**ND (n)**
Pharmacologically treated hypertension	2941	-
	(59.1%)	
Previus myocardial infarct	554	156
	(11.5%)	(3.1%)
Previous stroke	426	27
	(8.6%)	(0.5%)
History of atrial fibrilation	875	302
	(18.7%)	(6.1%)
Coronary heart disease (without myocardial infarct)	696	173
	(14.9%)	(3.5%)
Congestive heart failure	531	184
	(11.1%)	(3.7%)
Pharmacologically treated diabetes	1006	982
	(25.2%)	(19.7%)
Pharmacologically treated dyslipidaemia	1258	-
	(25.3%)	
Current smoker	449	21
	(9.1%)	(0.4%)
Overweight/obesity	3337	355
(BMI ≥ 25 kg/m^2^)	(72.2%)	(7.1%)

*ND* – not documented.

**Table 3:** Frequency of use of OAP and/or OAC in association with the cardiovascular risk factors

**Risk factor**	**OAP or OAC**	**OAP**	**OAC**	**OAP and OAC**
	**n (%)**	**n (%)**	**n (%)**	**n (%)**
Pharmacologically treated hypertension	**1480**	1354	151	25
	**(50.32%)**	(46.04%)	(5.13%)	(0.85%)
Previus myocardial infarct	**375**	350	35	10
	**(67.69%)**	(63.18%)	(6.32%)	(1.81%)
Previous stroke	**261**	237	25	1
	**(61.27%)**	(55.63%)	(5.87%)	(0.23%)
History of atrial fibrilation	**498**	396	117	15
	**(56.91%)**	(45.26%)	(13.37%)	(1.71%)
Coronary heart disease (without myocardial infarct)	**426**	382	58	14
	**(61.21%)**	(54.89%)	(8.33%)	(2.01%)
Congestive heart failure	**341**	290	61	10
	**(64.22%)**	(54.61%)	(11.49%)	(1.88%)
Pharmacologically treated diabetes	**471**	432	43	4
	**(46.82%)**	(42.94%)	(4.27%)	(0.40%)
Pharmacologically treated dyslipidaemia	**795**	726	86	17
	**(63.20%)**	(57.71%)	(6.84%)	(1.35%)
Current smoker	**132**	123	10	1
	**(29.40%)**	(27.39%)	(2.23%)	(0.22%)
Overweight/obesity	**1278**	1159	142	23
(BMI ≥ 25 kg/m^2^)	**(38.30%)**	(34.73%)	(4.26%)	(0.69%)

Data presented as n (%).

*OAP* - Oral antiplatelet drugs, *OAC* - Oral anticoagulant drugs.

Among all of the cardiovascular risk factors, hypertension was most associated with OAP treatment, a history of atrial fibrillation was most associated with OAC treatment, and coronary heart disease and male sex were most associated with combined OAP and OAC therapy (Table 4).

**Table 4:** Association of cardiovascular risk factors with OAP and/or OAC treatment

**Risk factor**	**OAP or OAC**	**OAP**	**OAC**	**OAP and OAC**
Pharmacologically treated hypertension	3.62	3.48	3.12	3.87
	(3 - 4.37)	(2.88 - 4.21)	(1.56 - 6.23)	(0.46 - 32.58)
	p < 0.01	p < 0.01	p < 0.01	NS
Previus myocardial infarct	2.53	2.67	0.71	2.19
	(1.93 - 3.31)	(2.06 - 3.45)	(0.43 - 1.19)	(0.8 - 5.95)
	p < 0.01	p < 0.01	NS	NS
Previous stroke	2.84	2.19	2.31	0.38
	(2.11 - 3.83)	(1.65 - 2.91)	(1.35 - 3.96)	(0.05 - 3.05)
	p < 0.01	p < 0.01	p < 0.01	NS
History of atrial fibrilation	1.54	0.95	9.01	1.64
	(1.25 - 1.89)	(0.77 - 1.16)	(5.74 - 14.14)	(0.59 - 4.57)
	p < 0.01	NS	p < 0.01	NS
Coronary heart disease (without myocardial infarct)	1.66	1.65	1.2	4.15
	(1.32 - 2.08)	(1.32 - 2.05)	(0.77 - 1.86)	(1.47 - 11.71
	p < 0.01	p < 0.01	NS	p = 0.01
Congestive heart failure	1.67	1.36	1.87	1.97
	(1.28 - 2.17)	(1.06 - 1.76)	(1.2 - 2.92)	(0.69 - 5.63)
	p < 0.01	p = 0.02	p = 0.01	NS
Pharmacologically treated diabetes	1.22	1.23	0.91	0.34
	(1.02 - 1.47)	(1.03 - 1.47)	(0.6 - 1.4)	(0.09 - 1.22)
	p = 0.03	p = 0.02	NS	NS
Pharmacologically treated dyslipidaemia	2.77	2.5	1.84	3.61
	(2.32 - 3.31)	(2.11 - 2.98)	(1.22 - 2.76)	(1.2 - 10.88)
	p < 0.01	p < 0.01	p < 0.01	p = 0.02
Current smoker	0.78	0.8	0.72	0.39
	(0.58 - 1.06)	(0.6 - 1.09)	(0.32 - 1.62)	(0.05 - 3.28)
	NS	NS	NS	NS
Previus smoker	0.92	0.97	0.61	0.32
	(0.76 - 1.11)	(0.8 - 1.17)	(0.38 - 0.97)	(0.11 - 0.94)
	NS	NS	p = 0.04	p = 0.04
Overweight/obesity	0.91	0.82	2.44	1.44
	(0.75 - 1.09)	(0.68 - 0.98)	(1.33 - 4.5)	(0.39 - 5.31)
(BMI ≥ 25 kg/m^2^)	NS	p = 0.03	p < 0.01	NS
Sex (male)	1.44	1.33	2.07	4.14
	(1.2 - 1.73)	(1.11 - 1.6)	(1.32 - 3.25)	(1.34 - 12.78)
	p < 0.01	p < 0.01	p < 0.01	p = 0.01

Data are presented as OR (95%CI) and p-value.

*OAP* - Oral antiplatelet drugs, *OAC* - Oral anticoagulant drugs.

Subsequently, we performed an analysis of probable indications for antiplatelet therapy. Among all of the patients treated with OAPs (n = 1648), this therapy was applied as secondary cardiovascular prevention (people with previous myocardial infarction, previous stroke, and history of coronary heart disease) in 717 respondents (43.5%). On the other hand, 705 people (42.8%) used OAP drugs in the primary prevention of cardiovascular disease. Among them, 58 respondents (3.5%) had no indications (SCORE <5%), 174 respondents (10.6%) had relative indications (SCORE ≥5% and <10%), and 473 respondents (28.7%) had definitive indications (SCORE ≥10% and/or atrial fibrillation). It was impossible to establish indications in 226 respondents (13.7%) (lack of data).

Among all the respondents treated with OACs (n = 165), 117 (71%) persons had a history of atrial fibrillation. Other indications for anticoagulant therapy could not be established in this group because of the lack of necessary data.

We also performed an analysis of optional indications for preventive therapy among respondents not treated with OAP or OAC drugs (n = 3192). Only 109 people (3.4%) had no indications for such therapy (SCORE <5), and 559 respondents (17.5%) had relative indications (SCORE ≥5 and <10). Secondary cardiovascular pharmacological prevention should be considered in 482 respondents (15.1%), and primary cardiovascular pharmacological prevention (SCORE ≥10 and/or atrial fibrillation) should be considered in 1447 respondents (45.3%). It was impossible to establish optional indications in 595 respondents (18.7%) (lack of data).

Among the patients with atrial fibrillation (n = 875), the CHADS_2_ score was calculated in 679 people (lack of data in 196 cases). Among the respondents with 0 points (n = 33), seven persons (21.2%) were treated (all of them with OAPs). Among the respondents with 1 point (n = 138), 47 persons (34.1%) used OAPs and 14 persons (10.1%) used OACs. Among the respondents with 2 or more points (n = 508), 254 persons (50%) received OAPs and 76 persons (15%) received OACs.

## Discussion

The percentage of elderly people in Poland (65 years and older) is currently 13.4%, and it has still been growing by approximately 2% over the last decade [14]. The PolSenior project was the first study in Poland involving such a large group of older people and concerning various aspects of their lives (health, social and economic). The study involved nearly 5,000 people aged 65 years and older, which constitutes approximately 1% of all Polish citizens in that age category.

Until the current study, there were no studies concerning the frequency of use of antiplatelet and anticoagulant drugs in the prevention of cardiovascular diseases in elderly people in Poland. Similar research in other countries is rare [15-18]. Our study showed that, in Poland, ASA is the most commonly used drug in prevention of thromboembolic events in elderly people. This medicine was regularly taken by as many as 32% of PolSenior respondents, both for primary and secondary prevention of cardiovascular disease. ASA use among adults aged 40 years and older in the United States is 41% (data from a nationally representative Internet-based survey involving 1299 adults) [18]. Other antiplatelet drugs were used less frequently in Poland, with ticlopidine used slightly more than clopidogrel (1.6% vs 1.14%), which might be attributed to the price of the drug. Until recently, the preparations of ticlopidine in Poland were much cheaper than the preparations of clopidogrel. It should be noted that despite similar mechanisms of action, these drugs significantly differ from each other. Ticlopidine is characterised by a slower time of action, making the drug useless in acute coronary syndromes, and is less convenient, with dosing twice a day. Clopidogrel acts much faster than ticlopidine, has less frequent side effects (e.g., gastrointestinal bleeding), and its once-daily dosing is better for the patient. Dual antiplatelet therapy was used by 90 respondents (1.81%). In Poland, the most common combination was ASA with ticlopidine.

It is interesting that dipyridamole was not used in Poland. This drug is recommended in combination with ASA for secondary prevention of cardiovascular disease in patients after stroke [19]. Some large randomised clinical trials have confirmed the effectiveness of such action [20]. However, pharmacoeconomics represents an obstacle for common use of such combination of drugs. According to data from the United States, the monthly cost of treatment with dipyridamole and ASA is much higher (~$122) than the monthly cost of treatment with ASA alone (less than $2) [21]. In Poland, the price, as well as the low availability of dipyridamole, pose a problem.

New antiplatelet drugs, such as ticagrelor, which reversibly binds the P2Y12 receptor and noncompetitively blocks adenosine diphosphate-induced platelet activation, were not commonly used in Poland when the study was performed. Therefore, no participant in our study had used them.

The most widely used OAC in Poland is acenocoumarol (more than 3% of respondents), which is in contrast to the United States and Western Europe, where warfarin is more popular. Until recently, warfarin was unavailable in Poland. Now it is prescribed more often. Warfarin was only used by 18 respondents (0.4%). New anticoagulants, such as dabigatran and rivaroxaban, were not used by the examined people.

The decision to begin therapy with an antiplatelet drug should be undertaken after assessing a global cardiovascular risk. Following the current European guidelines, ASA is recommended in all patients with an established cardiovascular disease (coronary heart disease, previous strokes, and previous myocardial infarctions) unless there are any contraindications [22]. In asymptomatic people, ASA should be considered when the risk of death from a cardiovascular disease is high (5% or greater in the next 10 years) [22]. SCORE tables are used to estimate this 10-year-long risk of death [12,22]. On the basis of the underlying risk factors, such as sex, age, systolic blood pressure, total cholesterol, and a history of smoking, the risk of death from cardiovascular causes in patients aged 40 and older can be determined. However, this scale is less suitable for older people because the estimated risk almost always exceeds 5% because of age (and sex), while other risk factors remain low [22]. This explains why, for the purposes of this study, we identified a group of people with a SCORE result between 5% and 9%, and considered that they had relative indications for primary cardiovascular preventive therapy. We believe that a SCORE result of 10% or higher better indicates the necessity for cardiovascular pharmacological prevention in older people [23].

We found that in Poland, antiplatelet drugs should be used more often. For example, 482 untreated respondents (15.1%) had a recognised cardiovascular disease and 1230 untreated asymptomatic respondents (38.5%) had an estimated SCORE ≥10%. After stroke, approximately 60% of people took medications, which is less than that in Western Europe [15], but is similar to the inhabitants of Asian countries [16]. Those with a history of myocardial infarction applied secondary prevention slightly more often than people after stroke (~70%).

All of the patients with atrial fibrillation had at least one risk factor (age ≥ 65 years); therefore, according to current guidelines, anticoagulant therapy should be considered for all these patients [24]. Our results showed that only 13% of the examined people with atrial fibrillation had used OACs, and slightly more than 40% used OAPs. This percentage appears to be inadequate, but we could not establish the reasons for omitting such a therapy (lack of data). It also appears that OACs were insufficiently used in patients after a stroke with concomitant atrial fibrillation (only one-fifth of patients). Analysis of CHADS2 score which estimates the risk of stroke in patients with atrial fibrillation confirms not adequate use of OACs in people with atrial fibrillation.

An important and common risk factor for cardiovascular disease is diabetes mellitus. Currently, recommendations for the use of antiplatelet drugs in diabetes have changed; they are similar to those for people without diabetes. Recent randomised clinical trials and meta-analyses have not confirmed the effectiveness of ASA in primary prevention of cardiovascular disease in diabetic patients. It has been shown that the use of ASA in people with diabetes and a low risk of cardiovascular disease (<5%) does not significantly affect the occurrence of cardiovascular events, but significantly increases the risk of bleeding complications (mainly haemorrhagic stroke and gastrointestinal bleeding) [25-28]. Among our respondents, 1399 people had diabetes, which constituted 28% of the whole study group. Less than 50% of them used OAP and/or OAC drugs.

The side effects of OAP and OAC therapy (mainly gastrointestinal and brain haemorrhage) should be considered. A benefit-to-risk ratio should be estimated in every patient [29]. The lack of data did not allow us to estimate such a ratio in the examined group of patients.

### Limitations of the study

A lack of data in some cases was an obstacle to estimate the indications for therapy in all of the respondents. Hypertension was recognised only in people treated with antihypertensive drugs, diabetes was recognised in people treated with insulin or oral drugs, and dyslipidaemia was recognised in people treated with statins or fibrates. Coronary heart disease and congestive heart failure were recognised only when they were documented (hospitalisation). The reasons for abandonment of therapy (contraindications) could not be established because of the lack of data.

The total responder rate in the study was rather low (42.58%; described in a previous paper) [11]. We presume that people who decided to take part in the project better adhered to medical recommendation and cared about their health more than those who refused. Therefore, the number of older people treated with OAP and/or OAC drugs in Poland could be even lower than originally described.

## Conclusions

Our study shows that the total percentage of treated elderly people with an indication for antiplatelet and/or anticoagulant therapy is too low in Poland. The most popular drug used in the prevention of cardiovascular diseases is ASA. The frequency of use of OAP and OAC drugs in elderly people is significantly affected by age, sex, place of residence, level of education, and personal income. Among the thienopyridine derivatives, ticlopidine is the most popular medication in Poland. OAC drugs are applied too rarely among older people with atrial fibrillation. Acenocoumarol is more popular than warfarin. Dipyridamole, as well as new anticoagulant drugs (e.g., dabigatran and rivaroxaban) and new antiplatelet drugs (e.g., ticagrelor), are not used in elderly people in Poland. It is necessary to develop educational programs among general practitioners concerning current recommendations for pharmacological cardiovascular prevention.

### Ethics approval

The PolSenior project was approved by the Bioethics Commission of the Medical University of Silesia in Katowice.

## Abbreviations

ASA: Acetylsalicylic acid; OAC: Oral anticoagulant drugs; OAP: Oral antiplatelet drugs; ACE inhibitor: Angiotensin converting enzyme inhibitor.

## Competing interests

The authors declare that they have no competing interests in relation to this manuscript.

## Authors’ contributions

BLR – conception and design of the study, analysis and interpretations of data, literature search, drafted the manuscript. KP – coordination of the work, analysis and interpretations of data, correction of drafted manuscript. M Sk – performed statistical analysis, correction of drafted manuscript. M Sw – analysis and interpretations of data, corrections of drafted manuscript. AMM - analysis and interpretations of data, literature search, helped in drafting the manuscript. All authors read and approved the final manuscript.

## Funding

Project founded by Ministry of Science and Higher Education No PBZ- MEiN-9/2/2006.

## Pre-publication history

The pre-publication history for this paper can be accessed here:

http://www.biomedcentral.com/1471-2261/12/98/prepub
